# 二次异基因造血干细胞移植治疗70例移植后复发恶性血液病患者的疗效及安全性

**DOI:** 10.3760/cma.j.issn.0253-2727.2023.06.003

**Published:** 2023-06

**Authors:** 婷婷 韩, 阳 刘, 瑶 陈, 圆圆 张, 海霞 付, 晨华 闫, 晓冬 莫, 峰蓉 王, 景枝 王, 伟 韩, 育红 陈, 欢 陈, 于谦 孙, 翼飞 程, 昱 王, 晓辉 张, 晓军 黄, 兰平 许

**Affiliations:** 1 北京大学人民医院，北京大学血液病研究所，国家血液系统疾病临床医学研究中心，造血干细胞移植治疗血液病北京市重点实验室，北京 100044 Peking University People's Hospital, Peking University Institute of Hematology, National Clinical Research Center for Hematologic Disease, Beijing Key Laboratory of Hematopoietic Stem Cell Transplantation, Beijing 100044, China; 2 郑州市第三人民医院血液科，郑州 450000 Hematology Department，the Third People's Hospital of Zhengzhou, Zhengzhou 450000, China

**Keywords:** 异基因造血干细胞移植, 恶性血液病, 复发, 更换供者, Allogeneic hematopoietic stem cell transplantation, Malignant hematological disease, Relapse, Donor change

## Abstract

**目的:**

评估二次异基因造血干细胞移植（二次移植）治疗移植后复发恶性血液病患者的疗效及安全性。

**方法:**

纳入1998年3月至2020年12月于北京大学血液病研究所行二次移植的70例恶性血液病患者，对其临床资料进行回顾性分析。

**结果:**

全部70例患者中男49例，女21例，二次移植时中位年龄为31.5（3～61）岁；其中急性髓系白血病（AML）31例，急性淋巴细胞白血病（ALL）23例，骨髓增生异常综合征（MDS）及其他16例；30例患者在二次移植时更换供者，40例未更换供者。首次移植后中位复发时间为245.5（26～2 905）d。1例患者二次移植后原发病持续未缓解未获得粒细胞植入，其余69例患者均获得粒细胞植入。62例（88.6％）患者获得血小板植入，更换供者组、未更换供者组血小板植入率分别为（93.1±4.7）％、（86.0±5.7）％（*P*＝0.636）。更换供者组、未更换供者组二次移植后巨细胞病毒（CMV）感染发生率分别为（64.0±10.3）％、（37.0±7.8）％（*P*＝0.053），Ⅱ～Ⅳ度急性移植物抗宿主病（GVHD）发生率分别为（19.4±7.9）％、（31.3±7.5）％（*P*＝0.227），移植后100 d移植相关死亡率（TRM）分别为（9.2±5.1）％、（6.7±4.6）％（*P*＝0.648），1年慢性GVHD累积发生率分别为（36.7±11.4）％、（65.6±9.1）％（*P*＝0.031）。二次移植后中位随访767（271～4 936）d，更换供者组、未更换供者组二次移植后2年累积复发率（CIR）分别为（52.6±11.6）％、（62.4±11.3）％（*P*＝0.423），总生存率分别为（28.3±8.6）％、（23.8±7.5）％（*P*＝0.643），无病生存率分别为（28.3±8.6）％、（22.3±7.7）％（*P*＝0.787）。二次移植前原发病完全缓解组（29例）、未缓解组（41例）移植后2年总生存率分别为（46.4±10.4）％、（11.0±5.2）％（*P*<0.001）。多因素分析显示，首次移植后早期复发（≤6个月）及二次移植前原发病未获得完全缓解是影响二次移植后复发、总生存和无病生存的独立危险因素。

**结论:**

更换供者对移植后复发恶性血液病患者二次移植的主要结局没有影响。

## Abstract (en)

**Objective:**

To investigate the role of donor change in the second hematopoietic stem cell transplantation (HSCT2) for hematological relapse of malignant hematology after the first transplantation (HSCT1).

**Methods:**

We retrospectively analyzed patients with relapsed hematological malignancies who received HSCT2 at our single center between Mar 1998 and Dec 2020. A total of 70 patients were enrolled [49 males and 21 females; median age, 31.5 (3–61) yr].

**Results:**

Forty-nine male and 21 female patients were enrolled in the trial. At the time of HSCT2, the median age was 31.5 (3–61) years old. Thirty-one patients were diagnosed with acute myeloid leukemia, 23 patients with ALL, and 16 patients with MDS or other malignant hematology disease. Thirty patients had HSCT2 with donor change, and 40 patients underwent HSCT2 without donor change. The median relapse time after HSCT1 was 245.5 (26–2 905) days. After HSCT2, 70 patients had neutrophil engraftment, and 62 (88.6％) had platelet engraftment. The cumulative incidence of platelet engraftment was (93.1±4.7)％ in patients with donor change and (86.0±5.7)％ in patients without donor change (*P*＝0.636). The cumulative incidence of CMV infection in patients with and without donor change was (64.0±10.3)％ and (37.0±7.8)％ (*P*＝0.053), respectively. The cumulative incidence of grade Ⅱ–Ⅳ acute graft versus host disease was (19.4±7.9)％ *vs* (31.3±7.5)％, respectively (*P*＝0.227). The cumulative incidence of TRM 100-day post HSCT2 was (9.2±5.1)％ *vs* (6.7±4.6)％ (*P*＝0.648), and the cumulative incidence of chronic graft versus host disease at 1-yr post-HSCT2 was (36.7±11.4)％ versus (65.6±9.1)％ (*P*＝0.031). With a median follow-up of 767 (271–4 936) days, 38 patients had complete remission (CR), and three patients had persistent disease. The CR rate was 92.7％. The cumulative incidences of overall survival (OS) and disease-free survival (DFS) 2 yr after HSCT2 were 25.8％ and 23.7％, respectively. The cumulative incidence of relapse, OS, and DFS was (52.6±11.6)％ *vs* (62.4±11.3)％ (*P*＝0.423), (28.3±8.6)％ *vs* (23.8±7.5)％ (*P*＝0.643), and (28.3±8.6)％ *vs* (22.3±7.7)％ (*P*＝0.787), respectively, in patients with changed donor compared with patients with the original donor. Relapses within 6 months post-HSCT1 and with persistent disease before HSCT2 were risk factors for OS, DFS, and CIR. Disease status before HSCT2 and early relapse (within 6 months post-HSCT1) was an independent risk factor for OS, DFS, and CIR post-HSCT2.

**Conclusion:**

Our findings indicate that changing donors did not affect the clinical outcome of HSCT2.

异基因造血干细胞移植（allo-HSCT）是治疗恶性血液系统疾病的有效方法，移植后血液病复发是影响移植疗效的主要原因[1]。目前恶性血液病移植后复发尚无标准治疗方案。二次异基因造血干细胞移植（二次移植）作为移植后复发的重要治疗手段[2]–[8]，疗效并不乐观，移植后5年总生存（OS）率仅为10％～30％[2],[9]–[10]。本中心以往采用二次移植治疗25例移植后复发的恶性血液病患者，中位随访时间9.1（2.0～131.1）个月，8例患者生存，总的OS率为30.9％[11]。影响二次移植疗效的因素众多，有研究认为首次移植后复发可能与供者的移植物抗白血病（GVL）效应不足有关，因此提出更换供者来增强移植后GVL效应，但也仅有少数国外研究报道且结论不一致[12]–[13]。本研究对在本中心接受二次移植的70例移植后复发恶性血液病患者进行回顾性分析，旨在评估二次移植治疗移植后复发恶性血液病的疗效及安全性。

## 病例与方法

一、病例

纳入1999年10月至2020年12月于北京大学血液病研究所首次移植后复发并接受二次移植的70例恶性血液病患者，对其临床资料进行回顾性分析。

二、预处理方案及GVHD预防

首次移植采用北京大学血液病研究所的常规预处理方案，二次移植预处理方案采用以全身放射治疗（TBI）为主的方案及以BU为主的方案。首次移植、二次移植的GVHD预防均采用环孢素A（CsA）加短程甲氨蝶呤（MTX）和霉酚酸酯（MMF）方案。急性移植物抗宿主病（aGVHD）和慢性移植物抗宿主病（cGVHD）诊断和分度见文献[14]。

三、植入标准

粒细胞植入是指中性粒细胞计数（ANC）>0.5×10^9^/L连续3 d，血小板植入是指PLT>20×10^9^/L连续7 d且脱离血小板输注。

四、随访

采用查阅门诊/住院病例及电话联系等方式进行随访。随访截止时间为2022年6月21日。

五、统计学处理

采用SPSS16.0软件进行数据分析，对于临床特征采用描述性统计学方法，组间比较采用卡方检验或Fisher精确检验；生存曲线采用Kaplan-Meier法绘制。多因素分析采用Cox回归模型。

## 结果

一、首次移植情况

入组恶性血液病的患者共70例（男49例、女21例），中位首次移植年龄为33（3～61）岁。70例患者首次移植一般情况见表1。

**表1:** 70例行二次造血干细胞移植恶性血液病患者的一般资料及首次移植情况

指标	更换供者组（30例）	未更换供者组（40例）	*P*值
性别［例（%）］			0.114
男	24（80.0）	25（62.5）	
女	6（20.0）	15（37.5）	
首次移植时年龄［岁，*M*（范围）］	32.5（10~59）	30.5（3~61）	0.809
基础疾病［例（%）］			0.050
ALL	13（43.3）	10（25.0）	
AML	14（46.7）	17（42.5）	
MDS及其他	3（10.0）	13（32.5）	
移植前疾病状态［例（%）］			0.132
CR	27（90.0）	30（75.0）	
NR	3（10.0）	10（25.0）	
供者类型［例（%）］			0.029
单倍体	12（40.0）	19（47.5）	
同胞相合	14（46.7）	21（52.5）	
无关	4（13.3）	0（0）	
预处理方案［例（%）］			0.18
Bu/Cy	15（50.0）	18（45.0）	
Bu/Cy+ATG	14（46.7）	18（45.0）	
Bu/Flu+ATG	1（3.3）	0（0）	
TBI/Cy	0（0）	3（7.5）	
Bu/Flu	0（0）	1（2.5）	

注 ALL：急性淋巴细胞白血病；AML：急性髓系白血病；MDS：骨髓增生异常综合征；CR：完全缓解；NR：未缓解；Bu：白消安；Cy：环磷酰胺；ATG：抗胸腺细胞球蛋白；Flu：氟达拉滨；TBI：全身放射治疗

二、二次移植情况

1. 一般情况：首次移植后原发病中位复发时间为261（26～2905）d，复发至二次移植的中位时间为152（10～2383）d，两次移植间隔中位时间为626（37～3226）d。二次移植预处理方案：59例患者以TBI为基础，11例以Bu为基础。在二次移植预处理中，更换供者组中1例患者更换为另一名全相合供者，其余患者均采用了ATG预防GVHD；未更换供者组中，20例采用原单倍体供者的患者中有8例未采用ATG预防GVHD。二次移植方案详见表2。

**表2:** 70例异基因造血干细胞移植后复发恶性血液病患者的二次移植方案

指标	更换供者组（30例）	未更换供者组（40例）	*P*值
二次移植前疾病状态［例（%）］			0.441
CR	14（46.7）	15（37.5）	
NR	16（53.3）	25（62.5）	
供者类型［例（%）］			<0.001
单倍体	27（90.0）	19（47.5）	
同胞相合	1（3.3）	21（52.5）	
无关	2（6.7）	0（0）	
二次移植前DLI［例（%）］			0.819
是	21（70.0）	29（72.5）	
否	9（30.0）	11（27.5）	
二次移植前DLI［次，*M*（范围）］	1（0~4）	1（0~6）	0.924
二次移植预处理方案［例（%）］			0.394
TBI为主	24（80.0）	35（87.5）	
Bu为主	6（20.0）	5（12.5）	
ATG应用［例（%）］			<0.001
是	28（9.3）	12（30.0）	
否	2（6.7）	28（70.0）	

注 CR：完全缓解；NR：未缓解；DLI：供者淋巴细胞输注；TBI：全身放射治疗；Bu：白消安；ATG：抗胸腺细胞球蛋白

2. 造血重建：1例患者二次移植后原发病持续未缓解，粒细胞未植入，于移植后62 d死于复发，其余69例均获得粒细胞植入，中位植入时间为13（9～26）d；62例（88.6％）患者获得血小板植入，中位植入时间为15（9～72）d，8例患者未获得血小板植入。二次移植后100 d血小板累积植入率为（91.0±3.7）％，更换供者组、未更换供者组血小板植入率分别为（93.1±4.7）％、（86.0±5.7％）（*P*＝0.636）。65例患者在二次移植后为完全供者嵌合状态，5例为混合嵌合状态。

3. 二次移植后CMV感染情况：二次移植后31例患者发生CMV血症，中位发生时间为移植后31（3～143）d，移植后100 d内CMV感染累积发生率为（45.3±6.0）％。更换供者组、未更换供者组CMV感染的发生率分别为（64.0±10.3）％、（37.0±7.8）％（*P*＝0.053）。

4. 二次移植后GVHD发生情况：70例患者中30例二次移植后发生aGVHD，100 d累积发生率为（46.9±6.3）％，中位发生时间为二次移植后43（21～97）d。17例患者发生Ⅱ～Ⅳ度aGVHD，移植后100 d内Ⅱ～Ⅳ度aGVHD累积发生率为（26.4±5.5）％，中位发生时间为二次移植后50（21～97）d。更换供者组、未更换供者组分别有5、12例患者在移植后100 d内发生Ⅱ～Ⅳ度aGVHD，两组Ⅱ～Ⅳ度aGVHD累积发生率差异无统计学意义［（19.4±7.9）％对（31.3±7.5）％，*P*＝0.227］。

26例患者在二次移植后发生cGVHD，更换供者组、未更换供者组分别为8、18例；轻度7例，中度12例，重度7例。二次移植后1年cGVHD累积发生率为（53.6±7.5）％，中位发生时间为141（101～192）d。未更换供者组二次移植后1年cGVHD累积发生率高于更换供者组［（65.6±9.1）％对（36.7±11.4）％，*P*＝0.031］。

5. 二次移植后累积复发率（CIR）、OS和无病生存（DFS）：二次移植后中位随访时间767（271～4936）d，70例患者中，二次移植前原发病完全缓解（CR）患者（29例）移植后首次评估仍为CR；二次移植前41例患者处于未缓解（NR）状态，二次移植后38例获得CR，3例二次移植后仍未获得CR，二次移植CR率为92.7％。除外3例持续NR的患者，70例患者在二次移植后有31例再次复发，二次移植后复发的中位时间为102（32～740）d。二次移植后2年OS率为（23.6±5.2）％，2年DFS率为（15.9±5.5）％。生存曲线见图1。

**图1:**
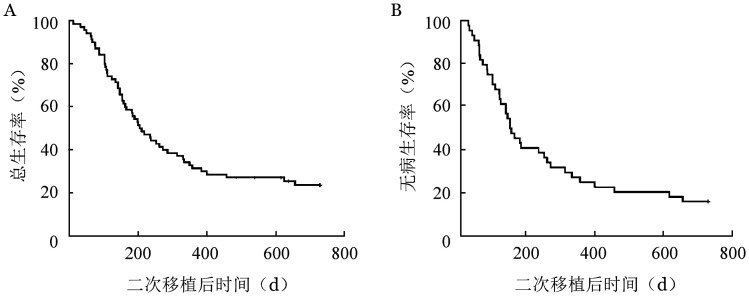
70例移植后复发恶性血液病患者二次移植后总生存曲线（A）和无病生存曲线（B）

更换供者组、未更换供者组二次移植后2年CIR分别为（52.6±11.6）％、（62.4±11.3）％（*P*＝0.423），OS率分别为（28.3±8.6）％、（23.8±7.5）％（*P*＝0.643），DFS率分别为（28.3±8.6）％、（22.3±7.7）％（*P*＝0.787）。二次移植前原发病CR、NR患者二次移植后2年OS率分别为（46.4±10.4）％、（11.0±5.2）％（*P*<0.001）。

在不同疾病类型中，二次移植时更换供者对预后也无影响。ALL患者中更换供者组与未更换供者组二次移植后2年OS率分别为（46.2±13.8）％、（13.3±12.0）％（*P*＝0.125），AML患者中更换供者组、未更换供者组二次移植后2年OS率分别为（14.3±9.4）％、（22.9±12.2）％（*P*＝0.197）。二次移植时，16例首次移植采用全相合供者的患者更换为单倍体供者，15例单倍体移植患者更换为另一单倍体供者，两组二次移植后2年OS率分别为（31.3±11.6）％、（33.3±13.6）％（*P*＝0.824）。本研究共有3例在复发时完成错配人类白细胞抗原基因组丢失（HLA-loss）检测，其中1例无HLA-loss，二次移植时更换供者，二次移植后死于复发；2例存在明显HLA-loss，二次移植更换供者，移植后1例死于CMV感染，1例死于血栓性微血管病（TMA）。

6. 死亡原因：至随访截止，51例患者死亡，其中31例死于原发病复发，12例死于感染，3例死于肝静脉闭塞病（HVOD），3例死于TMA，1例死于脑出血，1例死于爆发性肝炎。更换供者组、未更换供者组二次移植后100 d内移植相关死亡率（TRM）分别为（9.2±5.1）％、（6.7±4.6）％（*P*＝0.602），移植后2年TRM分别为（40.6±10.3）％、（40.1±10.7）％（*P*＝0.684）。

7. 危险因素分析：单因素分析显示，影响二次移植后OS的危险因素是首次移植前疾病状态（NR）、首次移植后早期复发（≤6个月）和二次移植前原发病未获CR。影响二次移植后DFS的危险因素是首次移植后早期复发（≤6个月）以及二次移植前原发病未获CR。将单因素分析中*P*<0.1的因素纳入多因素分析，结果显示，首次移植后早期复发（≤6个月）及二次移植前原发病未获CR是二次移植CIR、OS和DFS的独立危险因素（表3）。

**表3:** 影响二次异基因造血干细胞移植主要结局的危险因素分析

影响因素	总生存率				无病生存率				累积复发率		
	*HR*	95%*CI*	*P*值		*HR*	95%*CI*	*P*值		*HR*	95%*CI*	*P*值
单因素分析											
首次移植前疾病状态	2.296	1.224~4.308	0.010		1.801	0.961~3.373	0.066		–	–	–
首次移植后复发时间	2.679	1.556~4.613	<0.001		4.018	2.282~7.074	<0.001		4.411	2.161~9.007	<0.001
二次移植前疾病状态	0.340	0.919~0.605	<0.001		0.356	0.200~0.632	<0.001		0.399	0.192~0.829	0.014
多因素分析											
首次移植后复发时间	2.460	1.428~4.238	0.001		4.305	2.396~7.738	<0.001		4.544	2.183~9.458	<0.001
二次移植前疾病状态	0.365	0.205~0.650	0.001		0.335	0.186~0.603	<0.001		0.390	0.185~0.825	0.014

## 讨论

二次移植对于移植后复发的恶性血液病是可供选择的治疗方式之一[2]–[6]。本研究中二次移植后除4例持续NR的患者，其余患者粒细胞全部植入，移植后CMV血症、aGVHD和cGVHD发生率与既往报道接近，且二次移植后TRM与既往研究报道也一致[11],[15]–[16]。在是否更换供者的亚组分析中，我们发现，更换供者组、未更换供者组在植入、CMV感染、aGVHD及TRM方面差异均无统计学意义，而cGVHD的发生率较低提示更换供者进行二次移植是安全的。

ATG在allo-HSCT中可以用来去除T细胞，降低移植后的排斥反应强度[17]–[18]。在标准的CsA联合MTX预防GVHD的基础上加用ATG已被广泛应用于allo-HSCT中预防GVHD[18]–[19]。既往多项研究均显示使用ATG可显著降低Ⅲ/Ⅳ度aGVHD和cGVHD的发生率[18]，据此我们推测本研究未更换供者组cGVHD发生率高可能与部分患者未接受ATG预防有关。

有研究显示，在采用CsA+MTX+ATG方案预防GVHD的患者中CMV感染的发生率较采用CsA+MTX者高2.36倍[20]，本中心既往数据也显示单倍体移植患者CMV感染发生率显著高于同胞全相合移植患者[21]。据此，我们推测本研究中更换供者组CMV感染略高于（*P*＝0.053）未更换供者组的可能原因与本研究中更换供者组二次移植中采用ATG的患者明显多于未更换供者组，且更换供者组二次移植以单倍体移植模式为主有关。

数十年前在ASH上曾有循证指南强调二次移植在一些临床研究中的作用[22]，那么二次移植对于allo-HSCT后复发的恶性血液病患者究竟有什么样的作用呢？迄今为止，尚没有大宗的临床病例研究分析其有效性。我们曾报道过所有经过移植后复发患者二次移植后OS率为30.9％，本组allo-HSCT后复发恶性血液病患者二次移植后的生存情况与我中心既往研究及文献[23]报道的疗效接近。

国际骨髓移植登记处（IBMTR）一项纳入41例更换供者二次移植的研究显示，更换供者并不能提高生存率[12]。Hosing等[10]的研究却发现更换供者二次移植有改善预后的趋势，Imus等[24]的研究发现二次移植使用位点不合的供者可以提高OS率[10],[24]。而Christopeit等[25]报告了一项多中心回顾性研究结果，共纳入179例患者，其中同胞相合供者移植75例，无关供者移植104例，影响二次移植预后的因素是首次移植后的缓解持续时间以及二次移植前疾病状态，更换供者并没有改善二次移植的OS。本研究也显示在首次移植6个月后复发且二次移植前获得CR的患者，2年OS率达55.5％，DFS率达48.2％。而更换供者与使用原供者的二次移植疗效相当，影响二次移植后OS及DFS的独立危险因素是首次移植后早期复发（≤6个月）及二次移植前疾病是否CR，与文献报道一致。

HLA-loss是近年来提出的allo-HSCT后白血病细胞免疫逃避和疾病复发的机制，粗略估计HLA不相合移植后复发的患者中有10％～30％的复发患者受者特异性HLA基因丢失因此而导致白血病细胞出现对供者GVL效应抵抗[26]。本研究中首次移植接受全相合供者移植，二次移植更换为单倍体供者，或者首次单倍体或无关供者更换为新的单倍体供者均未影响移植疗效。本组经过化疗+DLI再次获得CR的患者可能对化疗药物敏感性以及对原供者的GVL效应都起到了筛选作用，这部分患者可能不存在HLA-loss，因此更换供者与否并未影响移植疗效。但是对于化疗+DLI无效的患者可能存在HLA-loss。由于本研究是回顾性研究，缺乏HLA-loss相关数据，更换的供者HLA位点情况无法回答，且HLA-loss仅为复发的可能机制之一，未来是否根据HLA-loss来选择二次移植供者进而改善移植预后尚需研究。

文献报道二次移植使用减低剂量预处理或非清髓预处理方案对于降低复发和TRM似乎没有帮助，OS与常规移植方案相当[27]–[29]。本组病例二次移植预处理采用含有TBI为主的方案以及以Bu为主的清髓预处理方案，单因素分析显示二次移植预处理方案对于移植预后没有影响。

综上，本组病例结果显示，首次移植后早期复发（≤6个月）和二次移植前原发病未获CR是影响二次移植疗效的主要因素，而更换供者以及预处理方式对二次移植疗效并无影响。
